# Bold-Independent Computational Entropy Assesses Functional Donut-Like Structures in Brain fMRI Images

**DOI:** 10.3389/fnhum.2017.00038

**Published:** 2017-02-01

**Authors:** James F. Peters, Sheela Ramanna, Arturo Tozzi, Ebubekir İnan

**Affiliations:** ^1^Department of Electrical and Computer Engineering, University of ManitobaWinnipeg, MB, Canada; ^2^Department of Mathematics, Adıyaman UniversityAdıyaman, Turkey; ^3^Department of Mathematics, Faculty of Arts and Sciences, Adıyaman UniversityAdıyaman, Turkey; ^4^Computational Intelligence Laboratory, University of ManitobaWinnipeg, MB, Canada; ^5^Department of Applied Computer Science, University of WinnipegWinnipeg, MB, Canada; ^6^Department of Physics, Center for Nonlinear Science, University of North TexasDenton, TX, USA

**Keywords:** fMRI, hypersphere, nucleus clustering, strong proximity, temporal pattern, Voronoï tessellation

## Abstract

We introduce a novel method for the measurement of information level in fMRI (functional Magnetic Resonance Imaging) neural data sets, based on image subdivision in small polygons equipped with different entropic content. We show how this method, called maximal nucleus clustering (MNC), is a novel, fast and inexpensive image-analysis technique, independent from the standard blood-oxygen-level dependent signals. MNC facilitates the objective detection of hidden temporal patterns of entropy/information in zones of fMRI images generally not taken into account by the subjective standpoint of the observer. This approach befits the geometric character of fMRIs. The main purpose of this study is to provide a computable framework for fMRI that not only facilitates analyses, but also provides an easily decipherable visualization of structures. This framework commands attention because it is easily implemented using conventional software systems. In order to evaluate the potential applications of MNC, we looked for the presence of a fourth dimension's distinctive hallmarks in a temporal sequence of 2D images taken during spontaneous brain activity. Indeed, recent findings suggest that several brain activities, such as mind-wandering and memory retrieval, might take place in the functional space of a four dimensional hypersphere, which is a double donut-like structure undetectable in the usual three dimensions. We found that the Rényi entropy is higher in MNC areas than in the surrounding ones, and that these temporal patterns closely resemble the trajectories predicted by the possible presence of a hypersphere in the brain.

## Introduction

In this paper, we introduce a novel technique of fMRI images analysis, called computational proximity method, i.e., nucleus clustering in Voronoï tessellations (Peters and Inan, 2016a). The images are subdivided in contiguous (without interstice or overlap) polygons, called the “Voronoï polygons.” They yield a density map, called “tessellation,” that makes it possible to make an objective measurement of the polygon areas' spatial distribution and helps to define “random,” “regular,” and “clustered” distributions (Duyckaerts and Godefroy, 2000; Edelsbrunner, 2014). Tessellations have been already used in neuroscience, i.e., to investigate spatial relations and connectivity between neural mosaics in the retina (Mozos et al., 2011) or to evaluate histological cortical sections (Peters et al., 2016) and pattern recognition (Hettiarachchi and Peters, 2017). In a Voronoï tessellation of an fMRI image, of particular interest is the presence of maximal nucleus clusters (MNC), i.e., zones with the highest number of adjacent polygons (Peters et al., 2016). MNC reveals regions of the brain, independent from blood-oxygen-level dependent (BOLD) signals, characterized by different gradient orientation and diverse functional dimensions (Saye and Sethian, 2011).

To evaluate the power and potentialities of this novel approach, we used the Voronoï tessellation technique combined with Rényi entropy, in order to test the brain-hypersphere hypothesis. Indeed, it has been recently hypothesized that brain activity is shaped in the guise of a functional hypersphere, which performs complicated 4D movements called “quaternionic” rotations (Tozzi and Peters, 2016a). They give rise to the so-called “Clifford torus,” a closed donut-like structure where mental functions might take place. The torus displays glued trajectories similar to a video game with spaceships in combat: when a spaceship flies off the right edge of the screen, it does not disappear but rather comes back from the left (Weeks, 2002). The human brain exhibits similar behavior, i.e., the unique ability to connect far-flung events in a single, coherent picture (Atasoy et al., 2016). During brain functions such as memory retrieval and mind-wandering, concepts flow from one state to another and appear to be “glued” together. It has also been recently proposed that the brain, when evaluated in the proper dimension (Kida et al., 2016), is equipped with symmetries in one dimension that disappear (said to be “hidden” or “broken”) in just one dimension lower (Tozzi and Peters, 2016b). A symmetry break occurs when the symmetry is present at one level of observation, but “hidden” at another level: it suggests that a 4D hypersphere could be equipped with symmetries, of great importance in order to explain central nervous system (CNS) activities, undetectable at the usual 3D cortical level.

Although we live in a 3D world with no immediate perception that 4D space exists at all, the brain hypersphere rotations can be identified through their “cross section” movements on a more accessible 3D surface, as if you recognized some object from its shadow projected on a screen. We may thus evaluate indirect clues of the undetectable fourth dimension, such hypersphere rotations' hallmarks or signs on a familiar 3D surface. Here we show that, in temporal fMRI series from spontaneous and evoked brain activity, MNC discloses the typical patterns of quaternionic rotations and hidden symmetries.

## Materials and methods

Here we describe the novel approach for fMRI neuroimages analysis. In brief, we show how fMRI images of the brain can be divided in small polygons, called tessellation regions, that display different levels of information. We elucidate how the differences amongst the small polygons make it possible for to evaluate otherwise hidden activities of the brain, and how such activities are the expression of transitory functional increases in nervous dimensions.

### Samples

In order to validate the novel method via systematic analysis, we favored studies focused on intrinsic (also called spontaneous, or resting-state) instead of task-evoked activity, because the former is associated with mental operations that could be attributed to the activity of a torus (donut-shaped view of brain activity): “screens” are glued together and the trajectories of thoughts follow the internal toroid-shaped surface of a hypersphere. For example, spontaneous brain activity has been associated with day dreaming propensities, construction of coherent mental scenes, autobiographical memories, experiences focused on the future, dreaming state (for a description of the terminology, see (Andrews-Hanna et al., 2014).

We evaluated two published data sets of spontaneous, intrinsic activity, for a total of 64 images:

- Spontaneous activity structures of high dimensionality (termed “lag threads”) can be found in the brain, consisting of multiple highly reproducible temporal sequences (Mitra et al., 2015). We retrospectively evaluated published video frames showing “lag threads” computed from real BOLD resting state rs-fMRI data in a group of 688 subjects, obtained from the Harvard-MGH Brain Genomics Superstruct Project. We assessed 4 sets of coronal sections (including a total of 54 Images) from the published videos (Threads 1, 2, 3, and 4): http://www.pnas.org/content/suppl/2015/03/24/1503960112.DCSupplemental
- We also analyzed 10 well-matched published pairs of networks, from the 20-component analysis of the 29,671-subject BrainMap activation database and (a completely separate analysis of) a 36-subject resting FMRI dataset (Smith et al., 2009).

Furthermore, we also evaluated 16 images from extrinsic (task-related) activity of the brain, in order to compare them with the intrinsic activity of the previous 64 images. We assesssed 16 fMRI images from visual tasks experiments, which illustrate the activity of different brain areas elicited by basic vision and object recognition (Mandelkow et al., 2016). In Mandelkow et al. (2016), a voxel-wise ANOVA F-statistic map (threshold *p* <1%, uncorrected) was superimposed on the T1 -weighted anatomical MRI of one representative subject (16 axial slices in radiological convention).

Each of the 80 tessellated images (64 from intrinsic and 16 from extrinsic brain activity) leads to the MNC mesh clustering described in the next paragraph. Such tessellations are also called tilings or meshes. This retrospective work was conducted in conformity with the ethical standards of the field and does not involve human subjects or animals.

### Tessellations of brain images

This section explains how fMRI brain images can be subdivided into small polygons. In technical terms, we introduce nucleus clustering in Voronoï tessellations of plane surfaces (Edelsbrunner, 2006; Peters, 2016). A Voronoï tessellation is a tiling of a surface with various shaped convex polygons. Let *E* be a plane surface such as the surface of an fMRI image and let *S* be a set of *generating points* in *E*. Each such polygon is called a Voronoï region *V*(*s*) of a

V(s)={x∈E:‖x−s‖≤‖x−q‖ for all q  in S}.

**Figure 1:**
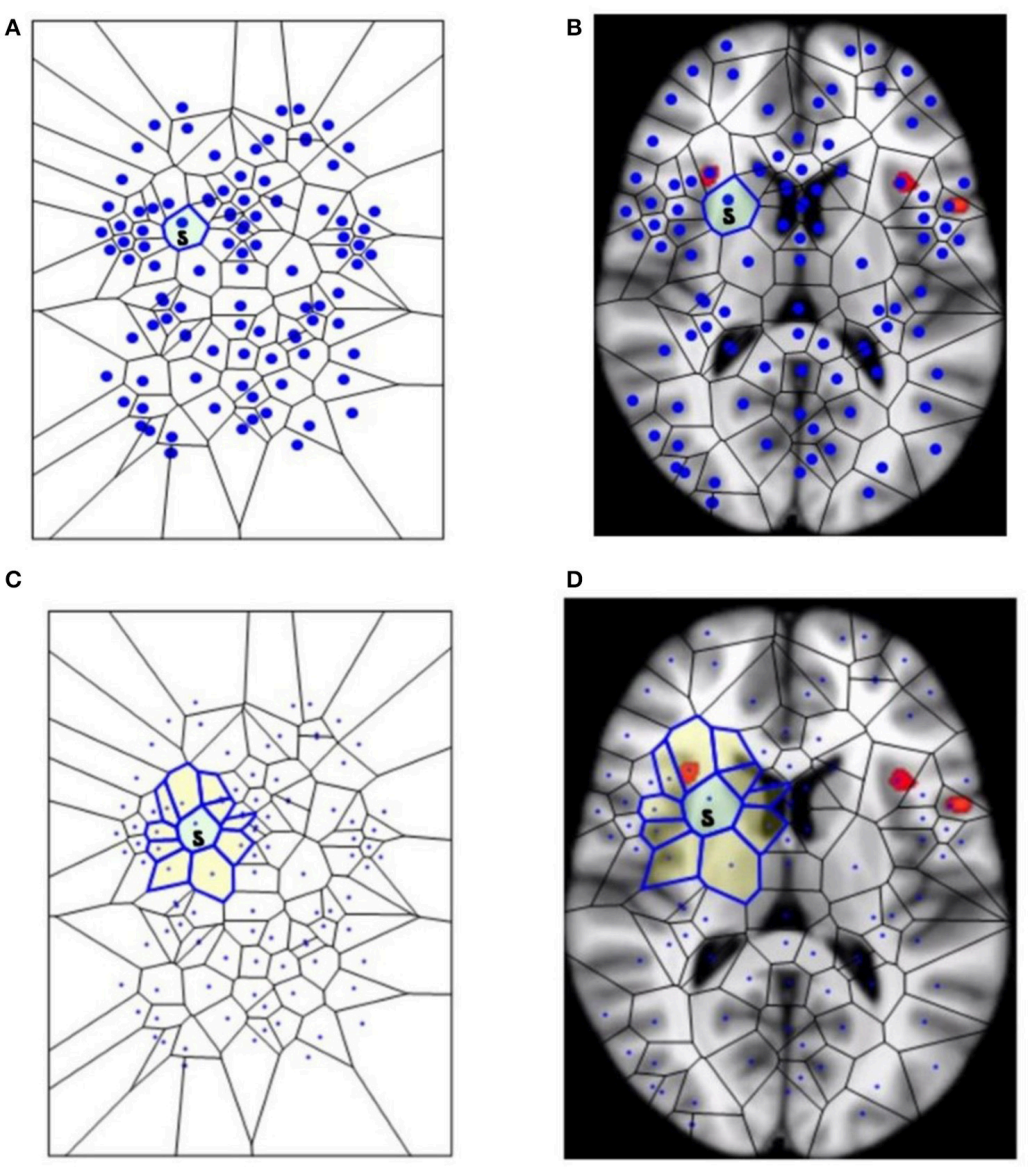
**The segmentation of fMRI images into small polygons, which makes it possible to compare the different morphological features displayed by the different groups. (A)** Surface tiling of a sample Voronoï region *V*(*s*) = 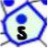. Each region in the tiling represents a generating point with particular fMRI image features, such gradient orientation and brightness. It is also worth noting that no two regions have the same description. For this reason, every Voronoï region has a slightly different shape. This tiling is derived from the fMRI image in **(B)**, which displays Voronoï region *V*(*s*) on a fMRI image taken from Mitra (11). **(C)** Displays a sample maximal nucleus cluster *N* for a particular generating point represented by the dot • in *N*. In this Voronoï tiling, the nucleus *N* has 10 adjacent polygons. Since N has the highest number of adjacent polygons, it is maximal. This cluster *N* is of particular interest, since the generating point • in *N* has *a gradient orientation that is different from the gradient orientation of any other generating point in this particular tiling*. In **(D)**, the maximal nucleus cluster *N* is shown *in situ* in the tiling of an fMRI image.

In other words, a Voronoï region *V*(*s*) is the set of all points *x* on the plane surface E that are nearer to the generating point *s* than to any other generating point on the surface (Figures 1A,B). In this investigation of fMRI images, each of the generating points in a particular Voronoï tessellation has a different description. Each description of generating point *s* is defined by the gradient orientation angle of *s*, i.e., the angle of the tangent to the point *s*.

### Nucleus clustering in tessellated images

This section elucidates why the small polygons described above are equipped with different, assessable characteristics. In very simple words, some polygons are different from the others, because they display higher number of sides. A *nucleus cluster* in a Voronoï tiling is a collection of polygons that are adjacent to (share an edge with) a central Voronoï region, called the cluster nucleus. In this work, the focus is on maximal nucleus clusters, which highlight singular regions of fMRI images. A pair of Voronoï regions are considered *strongly near*, provided the regions have an edge in common (Peters, 2016; Peters and Inan, 2016b). A *maximal nucleus cluster N* contains a nucleus polygon with the highest number of strongly near (adjacent) Voronoï regions (Figures 1C,D).

For technical readers, the gradient orientation angle θ of a point (picture element) in an fMRI image is found in the following way. Let *img*(x,y) be a 2D fMRI image. Then:

$$\begin{array}{l} G_{x}=\frac{\partial img}{\partial x},\\ G_{y}=\frac{\partial img}{\partial y},\\ \theta ={\text{tan}}^{-1}\left[\frac{G_{y}}{G_{x}}\right]={\text{tan}}^{-1}\left[\frac{\frac{\partial img}{\partial y}}{\frac{\partial img}{\partial x}}\right]=\text{arctan}\left[\frac{\frac{\partial img}{\partial y}}{\frac{\partial img}{\partial x}}\right]. \end{array}$$

In other words, the angle θ of the generating point of mesh nucleus is the arc tangent of the ratio of the partial derivatives of the image function at a particular point (x,y) in an fMRI image.

In sum, for each fMRI temporal frame, we produced tessellated images with one or more maximal mesh regions (i.e., a maximal region which contains the maximal number of adjacent regions). Furthermore, we produced tessellated images showing one or more MNC. Each maximal nucleus cluster *N* contains a central Voronoï region—the nucleus—surrounded by adjacent regions, i.e., Voronoï region polygons.

**Figure 2:**
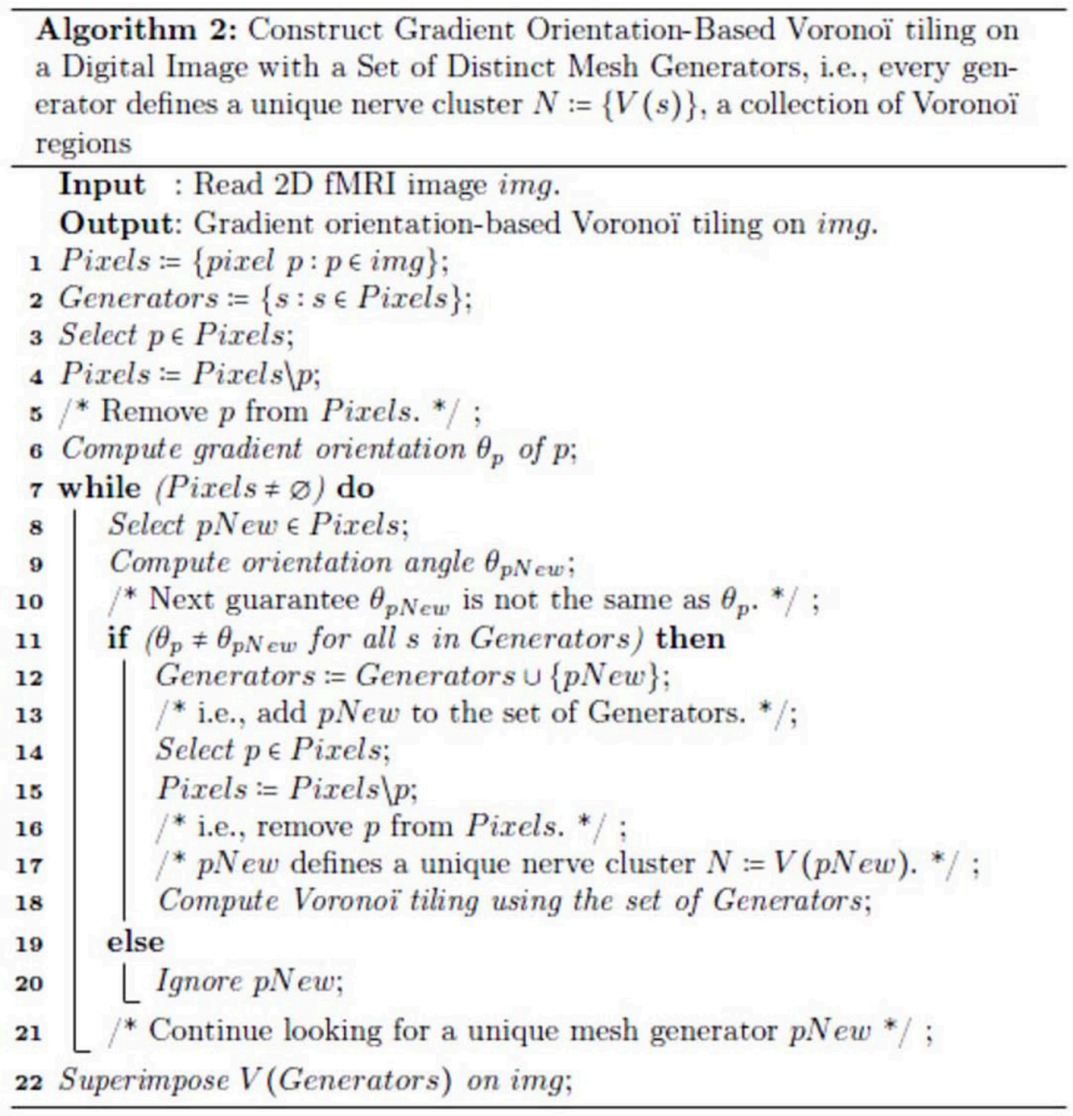
**The steps in the method used to construct the mesh on an fMRI image shown in Figure 1D**.

Also, for technical readers, we provide the steps for constructing a Voronoï tiling (regions) of an fMRI image, so that every generating point has gradient orientation (GO) angle θ that is different from the GO angles of each of the other points used in constructing the tiling on an fMRI image (see Figure 2). This form of construction guarantees that each nucleus of a mesh cluster is derived from a unique generating point. This is accomplished by weeding out all image pixels with non-unique GO angles. The end result is a collection of Voronoï regions that highlight different structures in a tessellated fMRI image. Each Voronoï region *V*(*s*) is described by feature vector that includes the GO of the generating point *s*.

Since each s is unique (not repeated in the set of generating points in Figure 2), each nucleus mesh cluster *N* has a unique description. Taking this a step further, we identify maximal nucleus clusters on a tessellated fMRI image. In effect, each maximal *N* tells us something different about each region of a tiled fMRI image, since we include, in the description of a maximal nucleus, the number adjacent regions as well as the GO of the nucleus generating point.

### Informational entropy in fMRI tessellations

Here we show why the fMRI polygons with more sides, called MNCs, display higher amounts of informational entropy, and therefore a higher informational content. Summarizing the previous paragraphs, the major new elements in the evaluation of fMRI images are: nucleus clusters, maximal nucleus clusters, strongly near maximal nucleus clusters, convexity structures that occur whenever maximum nucleus clusters intersect (Peters and Inan, 2016a). We showed in the above paragraphs that in a Voronoï tessellation of an fMRI image, of particular interest is the presence of ***maximal nucleus clusters*** (MNC), i.e., clusters with the highest number of adjacent polygons. In this section, we introduce a measure of the information that MNCs in fMRI images yield. We demonstrate that MNC reveal regions of the brain with higher levels of cortical information in comparison with non-MNC cortical regions, uniformly yield less information.

In a series of papers, Rényi (1961, 1966), introduced a measure of information of a set random events. Let *X* be a set random events such as the occurrence of polygonal areas in a Voronoï tessellation and let β > 0, β ≠ 1, *p*(*x*) the probability of the occurrence of *x* in *X*. Then Rényi entropy *H*_β_(*X*) is defined by

$$\begin{array}{l} \;X=\left\{x_{1},\cdots ,x_{n}\right\},\\ H_{\beta }(X)=\frac{1}{1-\beta }\log_{2}\sum_{i=1}^{n}p^{\beta }\left(x_{i}\right). \end{array}$$

Because of the relationship between Rényi entropy of a set of events and the information represented by events, Rényi entropy and information are interchangeable in practical applications (Rényi, 1982; Bromiley et al., 2010). In fact, it has been shown that Rényi entropy *H*_β_(*X*) is a monotonic function of the information associated with *X*. This means that Rényi entropy can be used as a measure of information for any order β > 0.

**Figure 3:**
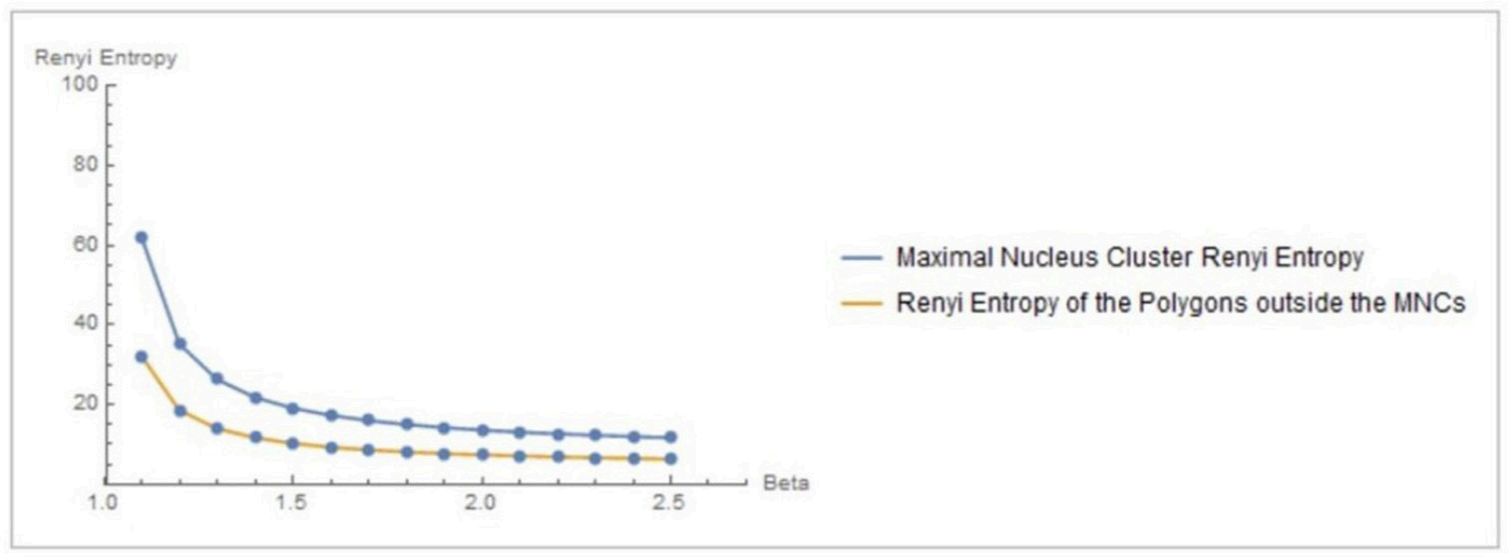
**Rényi entropy values of maximal nucleus clusters, compared with the surrounding areas of fMRI images**. The *x* axis displays the values of the Beta parameter for 1.1 ≤ β ≤ 2.5.

**Figure 4:**
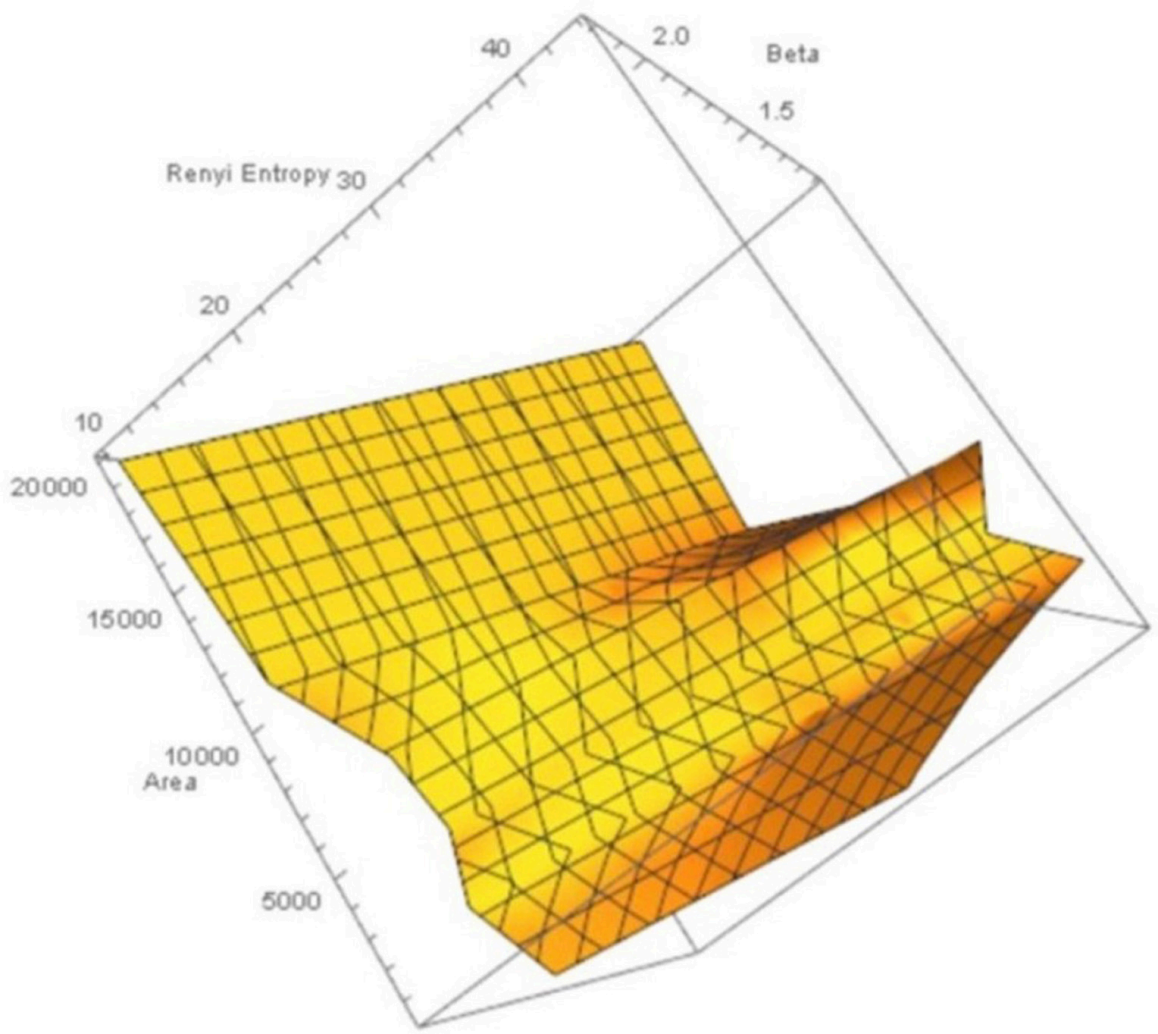
**Rényi entropy values vs. number polygon areas vs. 1.1 ≤ β ≤ 2.5 of maximal nucleus clusters in fMRI images**. MNC Nuclei surrounded by polygons with smaller areas have higher Rényi entropy, which tells us that smaller MNC areas yield more cortical information than MNCs with larger areas.

Let *X*_*MNC*_, *X*_*nonMNC*_ be sets of MNC polygonal regions and non-MNC polygonal regions in a random distribution of tessellation polygons. Also, let $p(x)=\frac{1}{x},p(y)=\frac{1}{y}$ be the probability of occurrence of *x* ∈ *X*_*MNC*_, *y* ∈ *X*_*nonMNC*_. Notice that the nuclei in MNCs have the highest concentration of adjacent polygons, compared to all non-MNC polygons. Based on measurements of Rényi entropy for observed MNC vs. non-MNC polygons, we have confirmed that Rényi entropy of nucleus polygon clusters is consistently higher than the set of non-MNC polygons (Figures 3, 4). This finding indicates that MNCs yield higher information than any of the polygon areas outside the MNCs.

In sum, Rényi entropy provides a measure of the information in maximal nucleus clusters and the surrounding regions of fMRI images. This means that the information from areas occupied by MNCs vs. non-MNC areas can be measured and compared. Furthermore, the maximal nucleus clusters are equipped with higher entropy values (and corresponding higher information), which contrasts with measure of information in the surrounding non-MNC regions. Hence, MNCs make it possible to pinpoint the sources of most information in fMRI images.

### Borsuk-Ulam theorem comes into play

Here we show how a simple theorem from topology is able to shed new light on the fMRI polygons equipped with different content of information. The Borsuk-Ulam Theorem (Borsuk, 1993; Dodson and Parker, 1997) states that:

$$\begin{array}{l} Every\;continuous\;map\;f:S^{n}\to R^{n}\;must\;identify\;a\;pair\\ \;of\;antipodal\;points\;(on\;S^{n})\\ \;with\;matching\;descriptions. \end{array}$$

That is, each pair of antipodal points on an *n*-sphere maps to Euclidean space *R*^*n*^ (Beyer and Zardecki, 2004). Points on *S*^*n*^ are *antipodal*, provided they are diametrically opposite (Marsaglia, 1972; Weisstein, 2015). For further details, see Tozzi and Peters (2016a,b). The two antipodal points can be used not only for the description of simple topological points, but also for more complicated structures (Borsuk, 1969), such spatial or temporal patterns functions, signals, movements, trajectories and symmetries (Saye and Sethian, 2011; Peters, 2016). If we simply evaluate CNS activity instead of “spatial signals,” BUT leads naturally to the possibility of a region-based, not simply point-based, brain geometry, with many applications (Tozzi and Peters, 2017). We are thus allowed to describe nervous systems functions or shapes as antipodal points on a n-sphere (Figure 3). This means that the activities under assessment (in this case, the 4D torus movements) can be found in the feature space derived from the descriptions (feature vectors) in a tessellated fMRI image.

**Figure 5:**
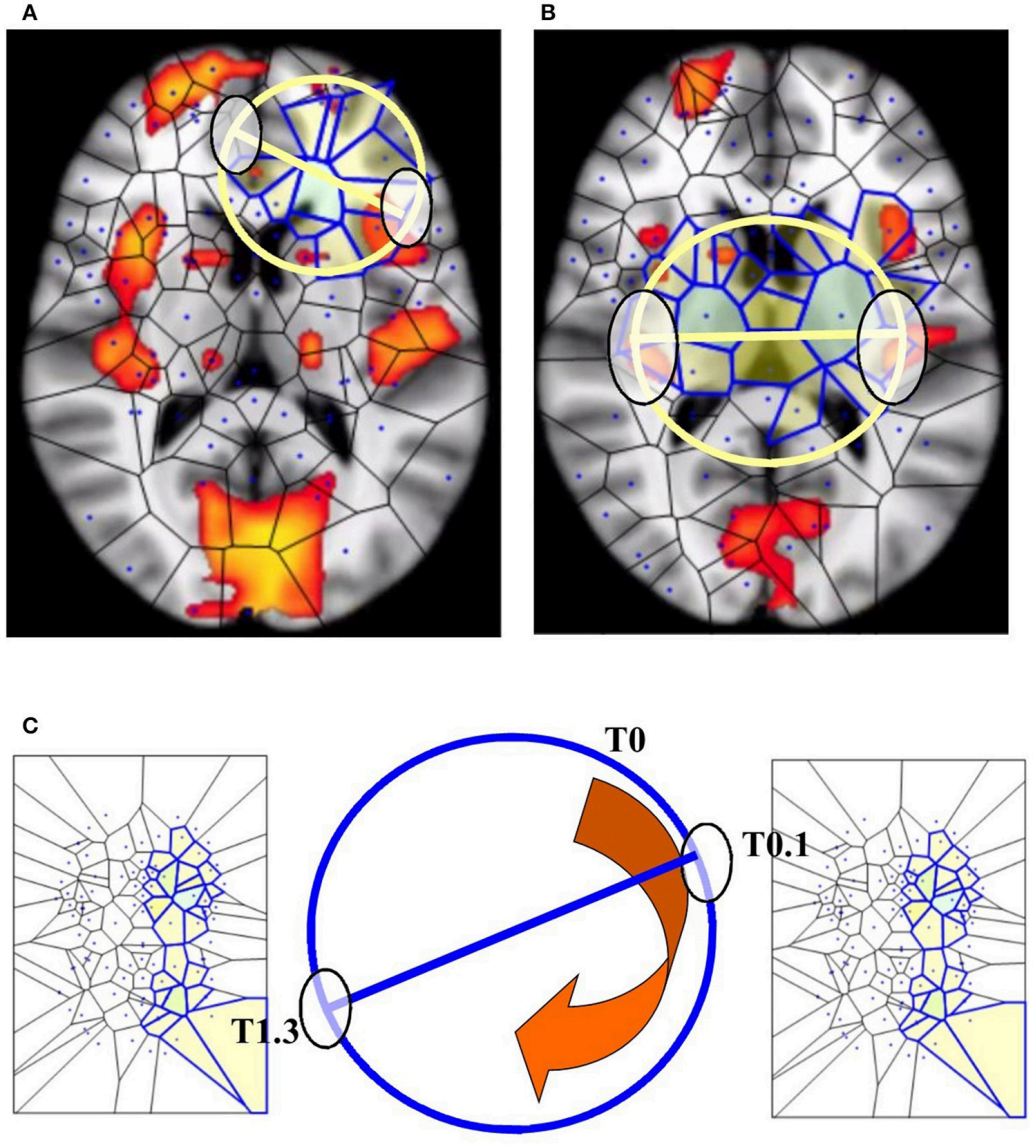
**BUT and its variants applied to fMRI neuroimages. (A,B)** display respectively one and two maximal nuclei clusters ((11), Thread 2). Antipodal points with matching description (on a spatial circumference) can be detected in both the images. Note that the MNC do not necessarily correspond to the “traditional” BOLD activations (shown in red) detectable in fMRI neuroimaging studies. **(C)** displays a temporal matching description between two maximal nuclei clusters at time T0.1 and T1.3 seconds. Note that, in this Figure, the n-sphere number n refers to the time, and not to a spatial dimension. The curved arrow depicts the time conventionally passing clockwise along the circumference of the n-sphere.

If we map the two antipodal points on a n–1 –sphere, we obtain a single point. The signal shapes' functions can be compared (Weeks, 2002; Saye and Sethian, 2011): the two antipodal points representing systems features are assessed at one level of observation, while the single point is assessed at a lower level (Figures 5A,B). Although BUT was originally described in terms of a natural number *n* that expresses a structure embedded in a spatial dimension, nevertheless the *n* value can stand for other types of numbers: it can be also cast as an integer, a rational or an irrational number (Tozzi and Peters, 2016b). We might regard functions or shapes as embedded in an *n*-sphere, where *n* stands for a temporal dimension instead of a spatial dimension. This makes it possible to use the *n* parameter as a versatile tool for the description of fMRI brain features (Figure 5C).

In sum, BUT and its variants say that:

- There exist regional spatial fMRI patterns (shapes, functions, vectors) equipped with matching description, e.g., they display the same features.
- We are allowed to assess the spatial patterns described by the MNC in terms of signals or temporal patterns (in our 4D case, the movements and trajectories on the 3D brain), in order to achieve a real-time description of the movements of the hypersphere.

### Quaternionic movements

In this paragraph, we describe the peculiar brain features that we expect our MNCs might help to elucidate and assess. In a previous study (Tozzi and Peters, 2016a), the presence of a hypersphere was detected invoking BUT: we viewed the antipodal points as brain signals opposite each other on a Clifford torus, i.e., we identified the simultaneous activation of brain antipodal signals as a proof of a perceivable “passing through” of the fourth dimension onto the nervous 3D surface.

**Figure 6:**
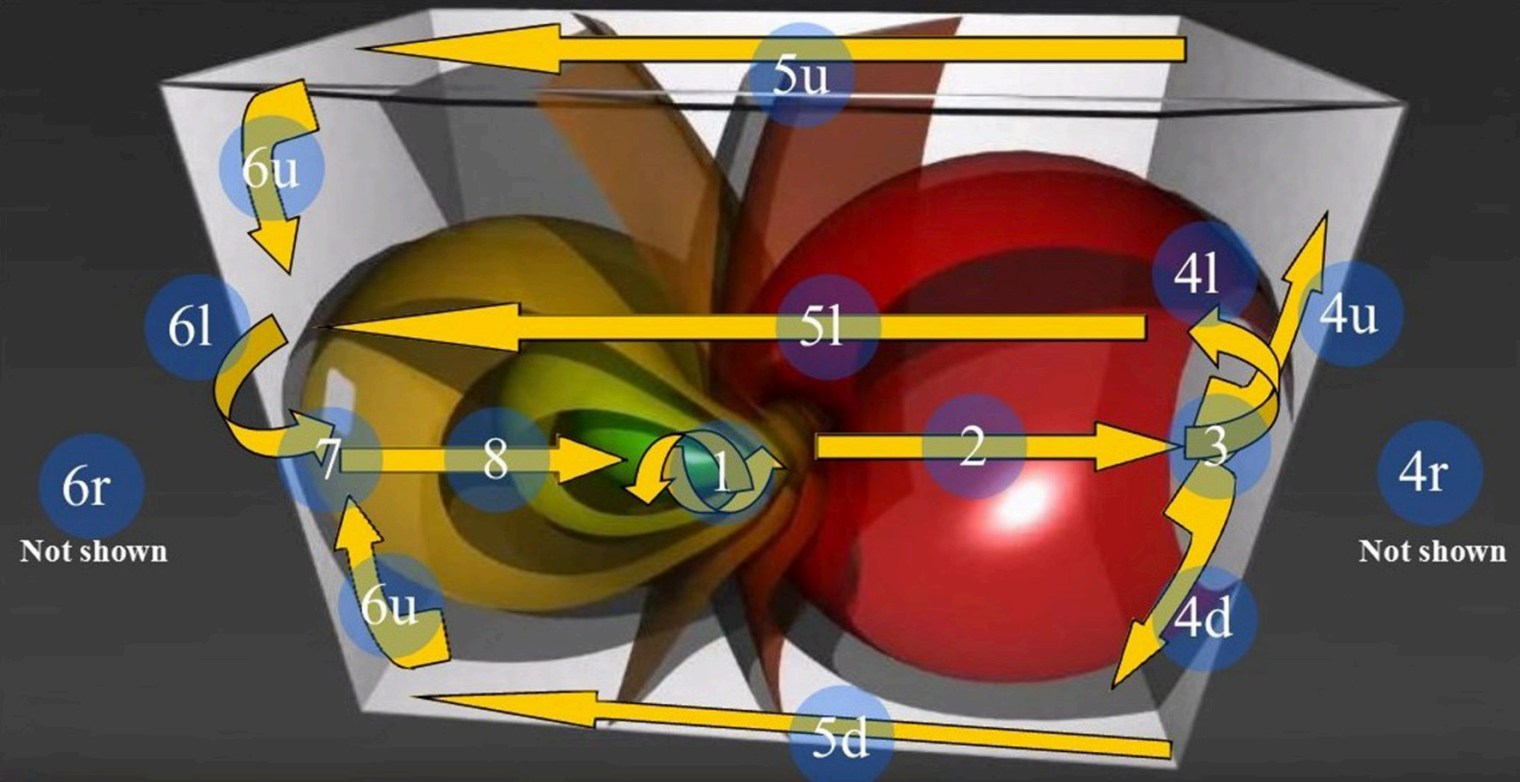
**The 3D Stereographic projection of the “toroidal parallels” of a hypersphere (from https://www.youtube.com/watch?v=QlcSlTmc0Ts; see also http://www.matematita.it/materiale/index.php?lang=en&p=cat&sc=2,745)**. The orange arrows illustrate the trajectories followed by the 4D quaternionic movements of a Clifford torus when projected onto the surface of the 3D space in which it is embedded. The circled numbers describe the trajectories, starting from the conventional point 1 (the letters u,d,r,l denote respectively the upper, down, right, and left trajectories on the surface of the 3D parallelepiped). Note that the arrows follow the external and medial surfaces of the 3D space in a way that is predictable. Just one of the possible directions of the quaternion movements is displayed: the flow on a Clifford torus may indeed occur in every plane. In this Figure, the spheres on the right grow in diameter, forming a circle of increasing circumference on the right surface of the 3D space. Conversely, on the opposite left side, the spheres shrink and give rise to a circle of decreasing circumference on the left surface of the 3D space.

Here we evaluated instead, in resting-state fMRI series, a more direct hallmark of the presence of a hypersphere: the trajectory and the temporal evolution of the signals on the 2D brain surface, in order to see whether they match the predicted trajectories of the Clifford torus. To evaluate a hypersphere in terms of a framework for brain activity, we first needed to identify potential brain signal loci where quaternion rotations might take place: we thus embedded the brain in the 3D space of a Clifford torus and looked for its hallmarks or hints (Figure 6).

## Results

In all the 80 assessed images, either from intrinsic or extrinsic activity of the brain, we found that clusters of higher activity, which are equipped with higher Rényi entropy compared with the surrounding zones, are scattered throughout different nervous areas. Therefore, some micro-areas of a specific anatomical zones contain more information than the adjacent ones. In other words, the MNC approach detects regions in the brain with the higher Rényi entropy, compared with the surrounding ones. In 50% of the 80 analyzed images, more than one cluster is detectable.

**Figure 7:**
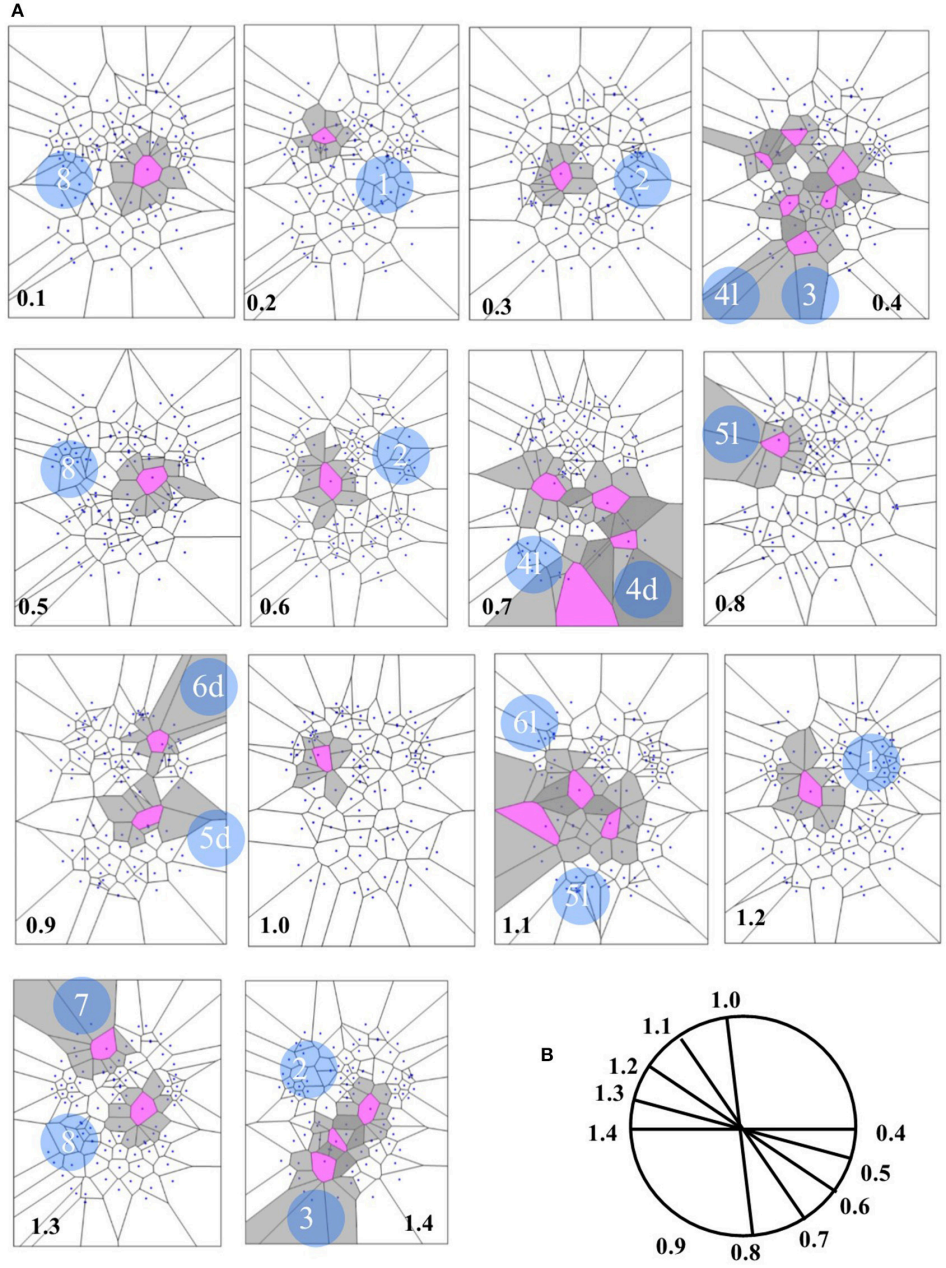
**(A)** Depicts a real pattern of maximal nuclei clusters temporal activation (from Mitra et al., 2015, Thread 4). Note that the typical trajectories of a Clifford torus are clearly displayed (see Figure 6 for comparison). If you look at the parallelepipedal 3D projections of the 4D quaternionic movements (Figure 6), the MNC embedded in the 2D brain surface stand for the 4D movements INTERNAL to the parallelepiped, while the MNC lying outside of the 2D brain surface stand for the 4D movements on the SURFACE of the parallelepiped in **(B)**. The movements described by maximal nucleus clusters are temporally specular. A matching description among temporal frames occurs, so that, for example, the frame 0.2 displays the same MNC features of 1.3. This means that the hypersphere moves in a stereotyped sequence and according a repetitive temporal sequence, following the trajectories predicted by the quaternionic model.

Concerning the 64 images from spontaneous, intrinsic, resting state activity of the brain, the data analysis shows also that the MNC activity displays in 79.68% (51 of 64) the typical features of the Clifford torus' movements. This supports the hypothesis that a functional hypersphere occurs during resting state brain activity (Figure 7). At the beginning, the trajectory of spontaneous activity follows the median sections (see timeframes 0.1–0.3 in Figure 7), then moves toward the posterior part of the brain, where a reflexion of four trajectories along the lateral surfaces occurs (0.4). This pattern closely matches the one predicted by the model illustrating the quaternionic movements on a Clifford torus. The temporal sequence also show the hypersphere moves on the brain, and it moves relatively slowly. The hypersphere does not display a regular or continuous movement, rather it proceeds forward and then backward for a short time (time 0.5 and 0.6). From 0.7, the trajectory follows the patterns predicted by Figure 6.

**Figure 8:**
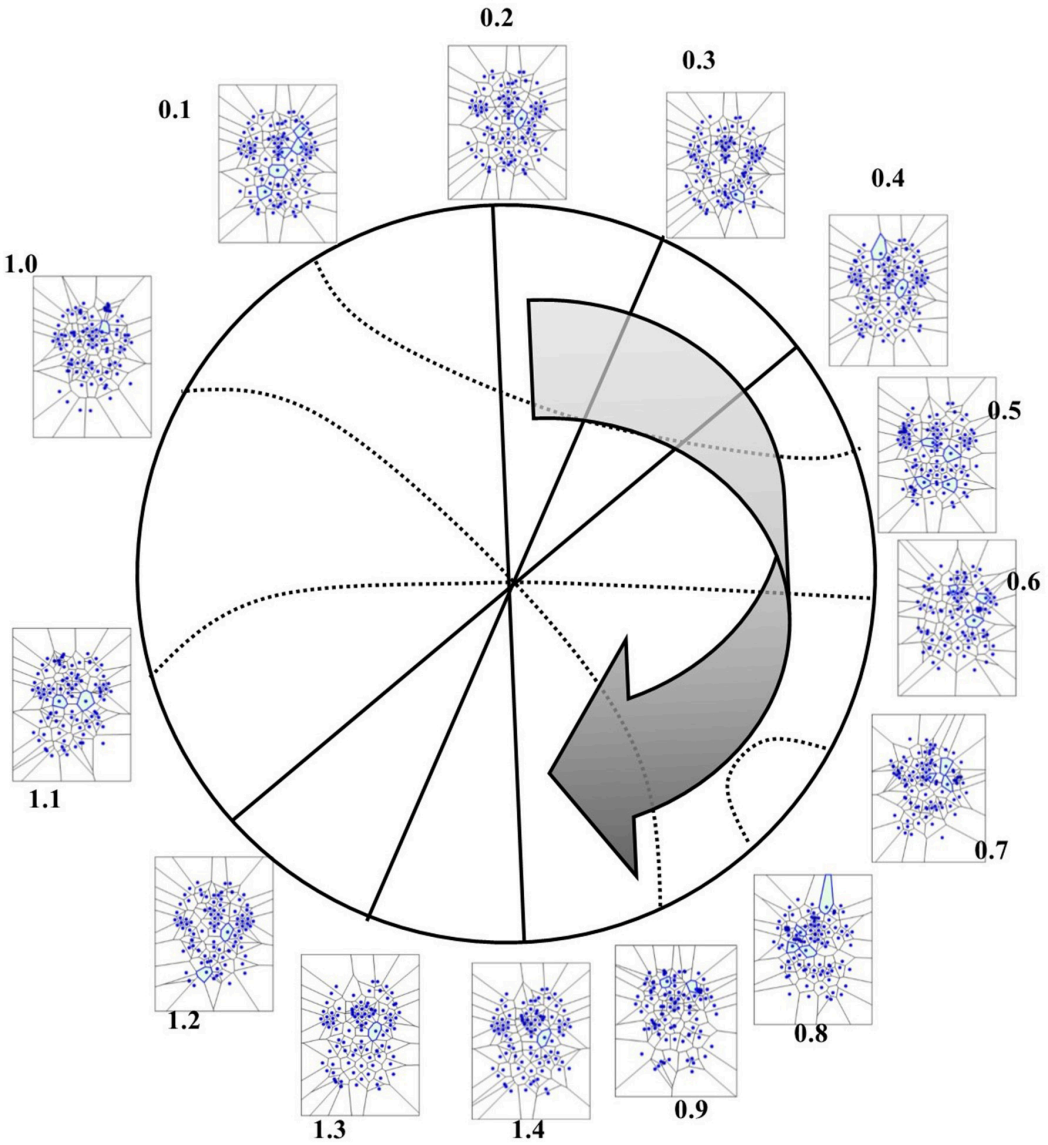
**Temporal antipodal points (from Thread 2 frames)**. The straight lines connecting opposite points on the temporal circumference depict “pure” antipodal points, while the curved lines depict non-antipodal points with matching description.

**Figure 9:**
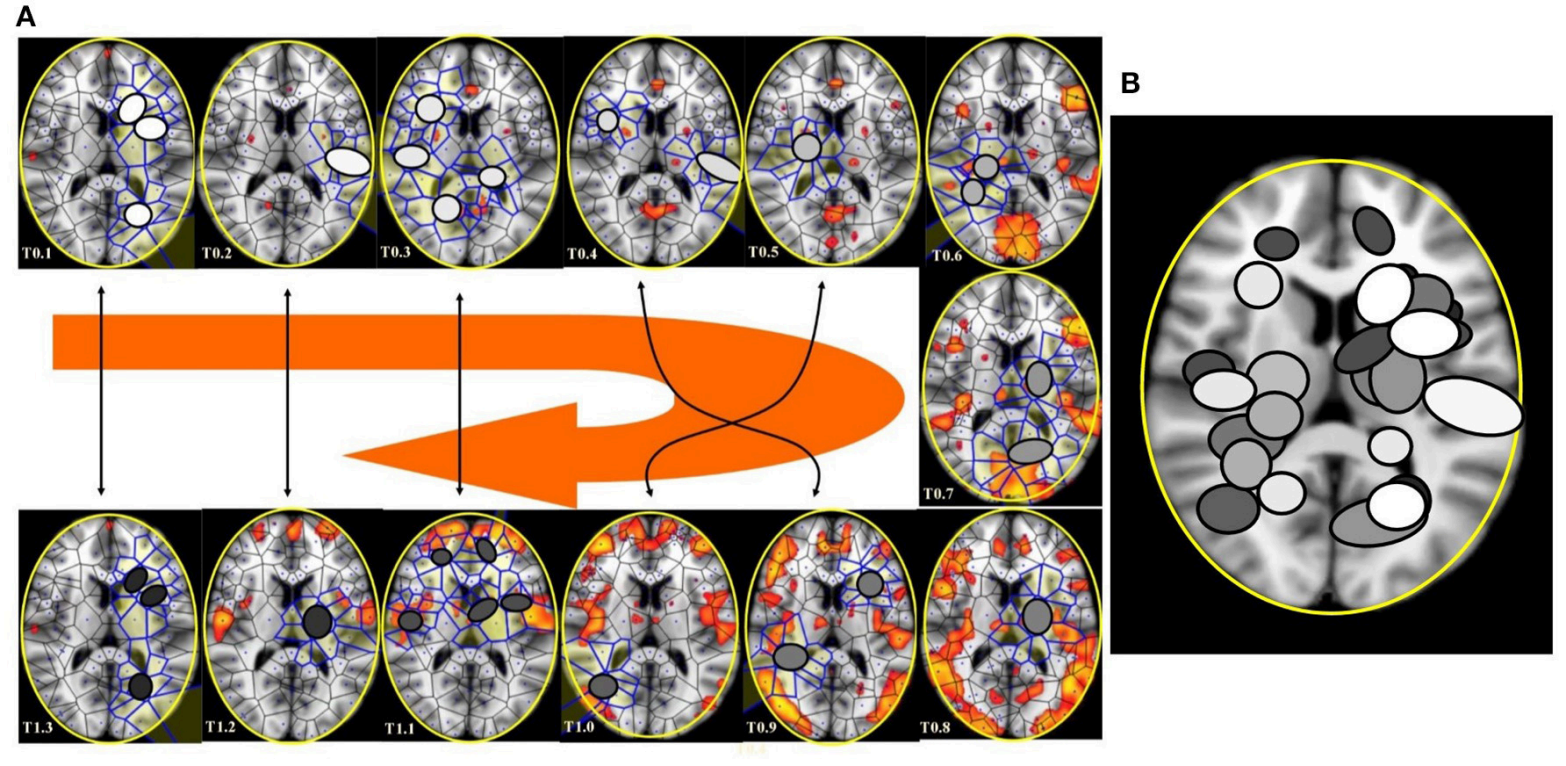
**(A)** This figure (from Thread 1 timeframes) depicts another way to illustrate temporal antipodal points. The temporal sequences are displayed clockwise, from *T* = 0.1 to *T* = 1.3. The Voronoï regions embedded in MNC are depicted as circles. The white circles refer to the presence of mesh clusters at high activity in the initial times, the gray circles in the intermediate times and the darker circles in the later times. **(B)** MNC activity on 13 superimposed, consecutive brain images from Thread 1 (from time T0 to T13). Note that the areas of nuclei activation are scarcely superimposed to the “classical” zones of BOLD activation (shown in red).

In the four available temporal series of the 54 images of spontaneous activity from Mitra et al. (2015), the MNC movements follow a specular, repetitive temporal pattern of activation (Figures 7–9). For example, the pictures of the first and the last time display MNC activity with matching description. Note that, in the 64 rest-related images, the areas encompassing MNCs do not match those equipped with BOLD activity in 71.87% (46/64) (Figure 9).

Concerning the 16 images from evoked, task-related activity elicited by basic vision and object recognition (Mandelkow et al., 2016), they showed a widely distributed MNC pattern throughout the brain. Fifty percent (8/16) of the images displayed the pattern predicted by the presence of a hypersphere, against the above mentioned 79.68% of correspondence detected in images of intrinsic activity.

The results can be summarized as follows: MNCs sequences of brain region activations, apart from differences depending on slight methodological distinctions among the assessed papers, exhibit a stereotyped topographical firing pattern. The movements of a hypersphere are clearly detectable, and the MNC activity clearly displays antipodal points in a temporal sequence, independent from fMRI activation. Such features are more marked in images taken from intrinsic, rather than extrinsic, task-related activity of the brain.

## Conclusions

There are two state-of-the-art approaches for understanding the communication among distributed brain systems using fMRI data. The first approach termed as *dynamic causal modeling* uses models of effective connectivity, while the second, called *Granger causal modeling*, uses models of functional connectivity (Friston, 2009, 2010). Our paper introduces a novel method, based on computational proximity (CP). This novelty can be expressed as follows: rather than being correlated with the “classical” BOLD activity, the CP method shows how spatial regions are correlated through their “proximity” (descriptive closeness to each other). From the experiments reported here, we are beginning to see different forms of brain function represented by the MNCs. We have demonstrated that computational proximity (i.e., strongly near nucleus mesh clusters) in 2D fMRI images is able to reveal hidden temporal patterns of Rényi entropy, enabling us to detect functional information from morphological data. The combination of tessellated images, topological framework provided by BUT, and Rényi entropy indicators of the information levels of image regions provide a systematic basis for the study of fMRIs. The validation of this approach to fMRI analysis stems from the mathematics of BUT and Rényi entropy. Central to this form of neural analysis is its introduction of tessellated fMRI images. Tessellation is a natural choice, since fMRI images by their nature both visual and geometric. There is a natural transition from a tessellated fMRI image to a consideration of those fMRI regions where the greatest changes occur. The greatest-change-regions are in the form of maximal nucleus clusters (MNCs) in each tessellated image. Detected MNCs in tessellated fMRI images are then subjected to analysis by BUT and the mechanics of Rényi entropy. The end result is a complete, verifiable, rigorous analysis fMRI images that are easily understood because of their visual character. The accuracy of the tessellated fMRI image approach stems from the computational geometry that leads to the detection of MNCs (Peters, 2016; Peters and Inan, 2016a; Peters et al., 2016; Tozzi and Peters, 2016b).

Here we have shown that a morphological analysis of simple 2D images taken from fMRI video frame sequences might give insights into the functional structure of neural processing. In a previous study, we evaluated the possible *hints* of a hypersphere on simple fMRI scans during resting state brain activity (Tozzi and Peters, 2016a). We showed how, due to the Borsuk-Ulam theorem (BUT), the fMRI activation of brain antipodal points could be a signature of 4D. The antipodal points predicted by BUT could be evaluated not just on images taken from fMRI studies, but also on datasets from other neuro-techniques, such as, for example, EEG. In the current study, we used a novel method, in order to confirm the data of the previous work with a completely different and more sophisticated approach. Indeed, looking at the sequences of maximal nucleus clusters and their entropy, we found experimental patterns compatible with the ones predicted by the hypersphere model. We detected on the 3D brain “shadows” of a 4D hypersphere rotating according to quaternion movements: these “hints” make it possible for us the possibility to visualize both the spatial arrangement and the movements of the corresponding Clifford torus. In other words, in particular during spontaneous brain activity, the apparently scattered temporal changes in MNCs follow a stereotyped trajectory which can be compared with the 4D movements of a hypersphere. Our study uncovered evidence of hypersphere during spontaneous activity, demonstrating that brain activity lies on a 3-sphere embedded in 4D space. How can be sure that the MNC reveals the presence of a brain hypersphere? Three cues talk in favor of this hypothesis. First, the MNC patterns, in particular during resting state fMRI data sets, closely resemble the theoretical trajectories predicted by Clifford torus movements. Second, temporal sequences of fMRI images display matching description, in agreement with the BUT dictates. Third, there is a difference in Rényi entropy between MNC and the surrounding zones, thereby pointing toward diverse levels of activity. The reproducibility of the hypersphere movements suggests that this organizational feature is essential to normal brain function. Because the Clifford torus incessantly changes its intrinsic structure, due to the different transformations of the quaternionic group, it is reasonable to speculate that each mental state corresponds to a different hypersphere's topological space. The concept of a hypersphere in the brain is also more noteworthy, if we frame it in the general picture of nervous symmetries (Tozzi and Peters, 2016b).

A shift in conceptualizations is evident in a brain theory of broken symmetries based on a hypersphere approach. It might be speculated that symmetries are hidden in a 3D dimension and restored in the higher dimension of the hypersphere, and vice versa. This means that brain functional and anatomical organization may be better assessed if one considers how certain hidden “symmetries,” essential to shaping brain gradients and activity, may only appear under the lens of higher-dimensional neural representations (Tozzi and Peters, 2016b), i.e., the hypersphere. We anticipate our essay to be a starting point for further evaluation, both in physiological and pathological conditions, of a neural fourth spatial dimension where other brain functions, such as perception, memory retrieval, might take place. It is also possible that every brain function displays a peculiar temporal pattern of such a novel entropic “activity.” In sum, we operationalized a novel, fairly inexpensive, image-analysis technique useful in detecting hidden temporal patterns in the brain. We can assess the spatial patterns described by MNCs in terms of entropy variations. While, by our “subjective” and “private” viewpoint, we tend to watch an image inferring the semantic parts in order to give it a meaning, MNCs allow the detection of the “objective” entropy, which do not necessarily correspond to the zones of the figure that we view as more significant. This means that MNCs provide a basis for quantifying high-yield information areas in fMRI image features that are normally “hidden” from our attention.

## Author contributions

All the authors equally contributed to: study concept and design, acquisition of data, analysis and interpretation of data, drafting of the manuscript, critical revision of the manuscript for important intellectual content, statistical analysis, obtained funding, administrative, technical, material support, and study supervision.

## Funding

The research has been supported by the Natural Sciences & Engineering Research Council of Canada (NSERC) discovery grants 185986 and 194376.

### Conflict of interest statement

The authors declare that the research was conducted in the absence of any commercial or financial relationships that could be construed as a potential conflict of interest.
